# Internet-based vestibular rehabilitation versus standard care after acute onset vertigo: a study protocol for a randomized controlled trial

**DOI:** 10.1186/s13063-022-06460-0

**Published:** 2022-06-16

**Authors:** Solmaz Surano, Helena Grip, Fredrik Öhberg, Marcus Karlsson, Erik Faergemann, Maria Bjurman, Hugo Davidsson, Torbjörn Ledin, Ellen Lindell, Jan Mathé, Fredrik Tjernström, Tatjana Tomanovic, Gabriel Granåsen, Jonatan Salzer

**Affiliations:** 1 Department of Clinical Sciences, Neurosciences, Umeå University, Umeå, Sweden; 2 Department of Radiation Sciences, Umeå University, Umeå, Sweden; 3 Department of Biomedical Engineering, Umeå University, Umeå, Sweden; 4 Sundsvall Regional Hospital, Sundsvall, Sweden; 5 Sollefteå Hospital, Region Västernorrland, Sollefteå, Sweden; 6 Department of Otorhinolaryngology, Head and Neck Surgery, Institute of Clinical Sciences, Sahlgrenska Academy, University of Gothenburg, Gothenburg, Sweden; 7 Department of Otorhinolaryngology, Head and Neck Surgery, Region Västra Götaland, Sahlgrenska University Hospital, Gothenburg, Sweden; 8 Department of Biomedical and Clinical Sciences, Linköping University, Linköping, Sweden; 9 Department of Otorhinolaryngology, Region Västra Götaland, Södra Älvsborg Hospital, Borås, Sweden; 10 Department of Clinical Neuroscience, Karolinska Institutet and Capio S:t Görans Hospital, Stockholm, Sweden; 11 Department of Clinical Sciences, Othorhinolaryngology, Lund University, Lund, Sweden; 12 Department of Clinical Science, Intervention and Technology, Karolinska Institutet, Stockholm, Sweden; 13 Department of Public Health and Clinical Medicine, Umeå University, Umeå, Sweden

**Keywords:** Acute onset vertigo, AVS, Internet-based rehabilitation, Online tool, Vestibular rehabilitation, Randomized controlled trial, RCT, Multicenter, Portable motion sensors, Gait function

## Abstract

**Background:**

Dizziness and vertigo affect around 15% of adults annually and represent common reasons for contacting health services, accounting for around 3% of all emergency department visits worldwide. Vertigo is also associated with excessive use of diagnostic imaging and emergency care and decreased productivity, primarily because of work absenteeism. Vestibular rehabilitation is an evidence-based treatment for chronic dizziness and supervised group exercise therapy has recently been shown to be effective after vestibular neuritis, a common cause of acute onset vertigo. However, such interventions are not readily available and there is a need for more easily accessible tools. The purpose of this study is to investigate the effects on vestibular symptoms of a 6-week online vestibular rehabilitation tool after acute onset vertigo, with the aim of aiding vestibular rehabilitation by presenting a more accessible tool that can help to reduce recovery time.

**Methods:**

Three hundred twenty individuals diagnosed with acute vestibular syndrome (AVS) will be recruited from multiple hospitals in Sweden and the effects of an online vestibular rehabilitation tool, YrselTräning, on vestibular symptoms after acute onset vertigo will be compared to standard care (written instructions leaflet) in a two-armed, evaluator-blinded, multicenter randomized controlled trial. The primary outcome will be the Vertigo Symptom Scale Short Form (VSS-SF) score at 6 weeks after symptom onset. Secondary outcomes include effects of the intervention on activities of daily living, mood and anxiety, vestibular function recovery, mobility measures, health economic effects, and the reliability of the Swedish VSS-SF translation.

**Discussion:**

Participants using the online vestibular rehabilitation tool are expected to recover earlier and to a greater extent from their symptoms as compared to standard care. Since up to 50% of people with AVS without treatment develop persistent symptoms, effective treatment of AVS will likely lead to a higher quality of life and help reduce the societal costs associated with dizziness and vertigo.

**Trial registration:**

Clinicaltrials.gov NCT05056324. Registered on September 24, 2021.

## Administrative information

Note: the numbers in curly brackets in this protocol refer to SPIRIT checklist item numbers. The order of the items has been modified to group similar items (see http://www.equator-network.org/reporting-guidelines/spirit-2013-statement-defining-standard-protocol-items-for-clinical-trials/).

**Table:** 

Title {1}	Internet-based vestibular rehabilitation versus standard care after acute onset vertigo: a study protocol for a randomized controlled trial
Trial registration {2a and 2b}.	Clinicaltrials.gov Identifier NCT05056324, September 24, 2021
Protocol version {3}	version 1.4, approved January 19, 2022
Funding {4}	Swedish Research Council grant, grant number 2020-00301.
Author details {5a}	Solmaz Surano M.D. Ph.D. ^1^ Helena Grip Ph.D. ^2, 3^ Fredrik Öhberg Ph.D. ^2, 3^ Marcus Karlsson M.Sc. ^3, 1^ Erik Faergemann M.D. ^1, 4^ Maria Bjurman P.T. ^5, 1^ Hugo Davidsson M.D. ^6, 7^ Torbjörn Ledin M.D. Ph.D. ^8^ Ellen Lindell M.D. Ph.D. ^6, 9^ Jan Mathé M.D. Ph.D. ^10^ Fredrik Tjernström M.D. Ph.D. ^11^ Tatjana Tomanovic M.D. Ph.D. ^12^ Gabriel Granåsen Ph.D. ^13^ Jonatan Salzer M.D. Ph.D. ^1^ 1. Department of Clinical Sciences, Neurosciences, Umeå University, Umeå, Sweden 2. Department of Radiation Sciences, Umeå University, Umeå, Sweden 3. Department of Biomedical Engineering, Umeå University , Umeå, Sweden 4. Sundsvall Regional Hospital, Sundsvall, Sweden 5. Sollefteå Hospital, Region Västernorrland, Sweden 6. Department of Otorhinolaryngology, Head and Neck Surgery, Institute of Clinical Sciences, Sahlgrenska Academy, University of Gothenburg, Gothenburg, Sweden 7. Region Västra Götaland, Sahlgrenska University Hospital, Department of Otorhinolaryngology, Head and Neck Surgery, Gothenburg, Sweden 8. Department of Biomedical and Clinical Sciences, Linköping University, Linköping, Sweden 9. Region Västra Götaland, Södra Älvsborg Hospital, Department of Otorhinolaryngology, Borås, Sweden 10. Department of Clinical Neuroscience, Karolinska Institutet and Capio S:t Görans Hospital, Stockholm, Sweden 11. Department of Clinical Sciences, Othorhinolaryngology, Lund University, Lund, Sweden 12. Department of Clinical Science, Intervention and Technology, Karolinska Institutet, Stockholm, Sweden 13. Department of Public Health and Clinical Medicine, Umeå University, Umeå, Sweden
Name and contact information for the trial sponsor {5b}	Sponsor and coordinating investigator: Jonatan Salzer, MD, PhD, Associate Professor, Consultant Neurologist, Department of Clinical Sciences, Neurosciences, Umeå University, Umeå, Sweden, jonatan.salzer@umu.se
Role of sponsor {5c}	The sponsor has full access to, and retains all rights to, the data generated in the study. The sponsor has no financial interests in the online vestibular rehabilitation tool.

## Introduction

### Background and rationale {6a}

Dizziness and vertigo represent common reasons for contacting health services and account for around 3% of all emergency department (ED) visits worldwide [1–5] and an estimated 15% of adults report a problem with dizziness in the past 12 months [6]. Otovestibular causes account for nearly one-third of all causes of acute dizziness in patients coming to the emergency room, while neurologic causes (including stroke) account for 11%. Acute unilateral vestibulopathy, migraine, benign paroxysmal positional vertigo (BPPV), and Ménière illness are the most common otovestibular causes [2]. In a study of sick leave in Sweden, vestibular disorders were found to be the most common cause of audio-vestibular causes of sick leave [7, 8]. Consequently, vertigo is associated with excessive use of diagnostic imaging and emergency care, in addition to decreased productivity primarily due to work absenteeism [9]. As such, there are economic implications associated with dizziness and vertigo where the full scope remains to be investigated.

Vestibular rehabilitation is an evidence-based treatment for chronic dizziness but is employed in less than 5% of target patients in primary care [10, 11]. Vestibular rehabilitation in the form of supervised group exercise therapy was recently shown to be effective in the rehabilitation of vestibular neuritis—a common cause of acute onset vertigo [12]. However, supervised group therapy interventions are not readily available, and the first line of treatment for vestibular rehabilitation is often written instructions. There is therefore a need for developing more accessible tools for vestibular rehabilitation.

An internet-based vestibular rehabilitation tool in English and Dutch has been developed and evaluated with chronic dizziness in primary health care [10, 13]. In the study using the Dutch version of the online vestibular rehabilitation tool on patients with chronic dizziness in primary care, the mean VSS-SF score at 3 months after symptom onset in the intervention group was 8.1 (SD 7.4), compared to 11.5 (SD 9.9) in the control group [13]. A change in the vertigo symptom scale short form (VSS-SF) score of ≥3 has been deemed as clinically significant [10, 11].

To date, internet-based vestibular rehabilitation has not been evaluated for the rehabilitation of acute onset vertigo despite data suggesting 30–50% of patients with acute onset vertigo have persisting symptoms 3 months after symptom debut [14]. In this intervention, we translate and validate this freely available vestibular rehabilitation tool (https://balance.lifeguidehealth.org/) and subsequently test, in a hospital-based cohort study, its effectiveness in preventing chronic vertigo, dizziness, and balance disturbances in patients with acute onset vertigo.

### Objectives {7}

The primary objective of this study is to assess the efficacy of the online vestibular rehabilitation tool, YrselTräning, on vestibular symptoms compared to standard written instructions after acute onset vertigo. We hypothesize that the group randomized to the online vestibular rehabilitation will experience significantly greater and faster balance recovery, as measured with the VSS-SF, compared with standard care. The full list of objectives is provided in Table 1 below.

**Table 1:** Full list of objectives

Primary objective	To compare the effect of the online vestibular rehabilitation tool with standard written instructions after acute onset vertigo on vestibular symptoms.
Secondary objectives	1. Compare the impact of the online vestibular rehabilitation tool with standard written instructions after acute onset vertigo on different aspects of everyday living.
	2. Compare how the online vestibular rehabilitation tool influences the walking ability compared with standard written instructions after acute onset vertigo.
	3. Compare how the online vestibular rehabilitation tool influences the lateral canal vestibulo-ocular reflex (VOR) recovery compared with standard written instructions after acute onset vertigo.
	4. Compare the long-term effects of early vs. delayed online vestibular rehabilitation on vestibular symptoms and mobility.
	5. Compare the effects of online vestibular rehabilitation with standard written instructions on vestibular rehabilitation compliance.
	6. Compare the health economic effects of online vestibular rehabilitation with standard written instructions.
	7. Compare the multi-joint kinematic output data from a portable multi-sensor movement analysis system, with the hip kinematic output data received from the mobile phone app.
	8. Translate and validate the VSS-SF scale from English to Swedish.
	9. Investigate the frequency of benign positional paroxysmal vertigo (BPPV) after AVS at different time points and using different evaluation methods; investigate the correlation between BPPV the DHI, VSS-SF, steps, and safety endpoints; and investigate the effect of treatment arm allocation on the risk for BPPV.
Safety endpoint:	The proportion of participants who have experienced falls/fractures since study start up until 6 weeks, 3 months, and 12 months; and the number of falls/fractures in each study arm at the same time points.

### Trial design {8}

This trial is designed as a randomized, controlled, evaluator-blinded, multicenter superiority trial with two parallel groups with an allocation ratio of 1:1. The primary endpoint is the VSS-SF score at 6 weeks. The standard care study arm will also be granted access to the online rehabilitation tool at the end of the core study at 3 months from randomization, meaning that if the online tool works better than standard treatment, all study participants will benefit from it. SPIRIT reporting guidelines have been used throughout this paper [15].

## Methods: participants, interventions, and outcomes

### Study setting {9}

Study participants will be recruited from various hospitals around Sweden: The University Hospital of Umeå, Sahlgrenska University Hospital, Karolinska University Hospital, Linköping University Hospital, Skåne University Hospital, Capio Saint Göran’s Hospital, Sollefteå Regional Hospital, Sunderby Hospital, Sundsvall Regional Hospital, Södra Älvsborg Hospital, and Östersund Regional Hospital.

### Eligibility criteria {10}

Participants will be 18 years old or over, have given written consent to participate in the study, have acute vestibular syndrome—i.e., new acute onset dizziness or vertigo within the previous 24-h period with pathological spontaneous or gaze-evoked nystagmus—and be symptomatic at inclusion. The full list of inclusion and exclusion criteria is available in Table 2.

**Table 2:** Inclusion and exclusion criteria

Inclusion criteria (all)	≥18 years old; *and*
	The individual has given written consent to participate in the study; *and*
	New acute onset dizziness or vertigo since ≥24 h with pathological spontaneous or gaze-evoked nystagmus (i.e., acute vestibular syndrome, AVS). The nystagmus as described above must be present at screening (between 24 h and 7 days from onset) spontaneously, gaze-evoked or head-shake-evoked, and documented; *and*
	Screening and inclusion within 7 days of onset of continuous symptoms; *and*
	Symptomatic at inclusion
Exclusion criteria (any)	Pre-existing vestibular disease or neurological disease anticipated to affect the ability to participate in the study or the effect of the intervention. N.B: Recurring AVS with no set diagnosis before inclusion is accepted, as is past transient neurological diseases such as TIA or migraine; *or*
	Inability to use the online rehabilitation tool, e.g., due to not having access to a computer, tablet, or smartphone, not having access to the internet or lacking in experience with such tools; *or*
	Mental inability, reluctance or language difficulties that result in difficulty understanding the meaning of study participation; *or*
	Medical and/or physical contraindications to making the required head movements (e.g., vertebral dissection) or otherwise participating in the training and testing exercises or data collection; *or*
	Medication or other substance intake which can affect the ability to participate in the study or the reliability of the measurement methods. These medications include regular use of anticonvulsants, antiemetics/motion sickness medications, benzodiazepines, and neuroleptics. Transient corticosteroid and/or antiemetic treatment related to the current vertigo is accepted.

### Who will take informed consent? {26a}

The local study coordinator, which includes physicians, nurses, and physiotherapists at care units, meets and briefly explains the study to potential study participants and asks whether they are interested to receive further information including written information about the study and to be screened for participation. The individuals are then screened for participation with a screening checklist (inclusion/exclusion criteria) including screening for nystagmus, using video Frenzel goggles (screening of eye movements looking straight forward, 30° to the right, 30° to the left, up, and down); the presence of nystagmus must be documented to be present at least 24 h after symptom debut. The full list of inclusion and exclusion criteria is available in Table 2.

The principal investigator (PI) or delegated staff at each site ensures that the participant is given full and adequate oral and written information about the study, its purpose, any risks and benefits, and the inclusion and exclusion criteria. The participant will also be informed that they can discontinue their participation at any time without having to provide a reason. Participants will be given an opportunity to ask questions and will be allowed time to consider participation. If the individual chooses to participate, both the participant and the investigator will sign the informed consent form before performing any study-specific activity in the study. A copy of the signed informed consent form and a copy of the study participation information will be provided to the participant. The participant will then be booked for an inclusion visit, which can take place the same day. The process will be documented in the participant’s source documents and the signed informed consents will be maintained with the essential documents. If new information becomes available that could have a substantial impact on a participant's future health and medical care, the affected participant(s) will be notified in writing. If new information is added to the study, the participant has the right to reconsider their continued participation.

### Additional consent provisions for collection and use of participant data and biological specimens {26b}

On the consent form, participants will be asked for permission to use of their data should they choose to withdraw from the trial. Participants will also be asked for permission for the research team to share relevant data with people from the Universities taking part in the research or from regulatory authorities where relevant. This trial does not involve collecting biological specimens for storage.

## Interventions

### Explanation for the choice of comparators {6b}

Individuals will be randomized to the intervention group, comprising an online 6-week vestibular training tool that demands 15–20 min of training each day; or the control group, consisting of written instructions.

The comparator (written instructions) was chosen because it is used as a first-line treatment for the target group by most participating sites. Furthermore, the alternative, physiotherapist-led group training sessions, is neither readily available nor practical to deliver in a cost-effective manner. No national guidelines or pre-existing decision-making support have been found on how the target group should be managed and treated in terms of vestibular rehabilitation.

### Intervention description {11a}

The participants recruited in this study will be randomized to:

1. Written instructions. The participants randomized to the standard study arm are given written instructions with the six different exercises from the online rehabilitation program and instructed to increase the difficulty if possible. Participants are also given general tips on how to prevent inactivity, secondary dizziness, and fear of motion. The data is collected in the same manner as for the intervention arm. Participants in the standard care study arm will be given access to the online rehabilitation tool at the completion of the core trial, which will take place 3 months after randomization, and will be followed until 12 months after randomization.

OR

2. Internet-based Vestibular Rehabilitation Tool, YrselTräning. The intervention consists of a digital tool (software) for guiding participants through a 6-week vestibular rehabilitation. Its main feature is that during the 6-week period, the digital tool gives participants customized exercises for each week depending on how they are progressing through scoring tests. It also keeps track of their progress and reminds the users when they should perform the exercises. The exercises are explained via text and video instructions. Since the intervention is a web application, it can be run on any platform that has an internet connection. Each user will be assigned a personal login for the tool. The online rehabilitation tool will only be used in the clinical investigation and according to the clinical investigation plan during the study. The sponsor provides the sites with written instructions (study documentation) and technical support.

The goal of the vestibular rehabilitation tool is to maximize central compensation for the vestibular and/or balance deficit. The program comprises daily workouts and weekly online sessions that consist of educational literature, symptom control approaches, scoring tests for discomfort, and exercise prescriptions for the following week. After each online session, the exercises for the following week will be tailored to the user and will become increasingly difficult with lower scores for discomfort, or vice versa if the user scores higher discomfort.

The vestibular rehabilitation tool contains 6 different exercises: (1) head shake with open eyes, where the gaze follows the direction of the head; (2) head nod with open eyes, where the gaze follows the direction of the head; (3) head shake with closed eyes; (4) head nod with closed eyes; (5) head shake with open eyes and fixated gaze; and (6) head nod with open eyes and fixated gaze. Depending on the symptom intensity, these exercises are performed sitting, standing, and walking (to promote safety the participants are instructed not to perform the walking exercises with their eyes closed). In addition, in order to cope with potential secondary anxiety issues related to vertigo, the tool includes 4 symptom control techniques: (1) controlled breathing; (2) relaxation; (3) stress management; and (4) thought control [16].

### Criteria for discontinuing or modifying allocated interventions {11b}

If a participant has a recurrence of acute vestibular syndrome within 6 weeks of inclusion, their participation in the study will be discontinued. The cause for the discontinuation is documented in the eCRF system's trial termination form, and any future data collection will be halted.

If a participant is unable to continue participating in the study (follow-up, data collection) owing to physical, mental, or technical issues, the site PI must be notified immediately. The trial termination form in the eCRF system is used to record the reason(s) for as well as the date of discontinuation.

If a participant is unable to continue participating in the study (training) owing to physical, mental, or technical issues, the individual will be asked to continue to participate in data collection and observation (ITT analysis).

### Strategies to improve adherence to interventions {11c}

Thorough manuals with step-by-step guidance have been prepared for the participants in both study arms. The participants in standard care arm are given a leaflet with detailed instructions and clarifying pictures to represent each vestibular rehabilitation exercise. The participants in the intervention arm are provided with a leaflet of log-in details and details of how to use the online application. Furthermore, the online application provides step-by-step instructions with clarifying videos showing participants how to perform the exercises. The information leaflets also provide participants with contact details of support staff should they have difficulty with the online application, where applicable.

### Relevant concomitant care permitted or prohibited during the trial {11d}

Although data supporting long-term effects is insufficient, Swedish guidelines recommend a short-term oral corticosteroid treatment for patients with vestibular neuritis [17, 18]. All participants with suspected vestibular neuritis (i.e., those with a uni-directional horizontal spontaneous or gaze-evoked nystagmus, a positive head impulse test in the opposite direction to the nystagmus, and no other neurological symptoms or findings) will be given corticosteroid treatment: 50 mg oral prednisolone daily for 5 days, and thereafter tapered in the following 5 days [18].

### Provisions for post-trial care {30}

Although we anticipate no harm from trial participation, any participant that suffers harm is entitled compensation under the Swedish Patient Injury Act. Since the study is performed under the Swedish public health care system, all participants are protected by the Swedish Patient Insurance provided by Landstingens Ömsesidiga Försäkringsbolag (LÖF).

### Outcomes {12}

To give thorough coverage of the potential effects of the online rehabilitation, several relevant aspects of subjective vertigo, everyday functioning, quality of life, mobility, balance, as well as objective physiological measurements of balance system recovery, are analyzed. The primary outcome variable is the vertigo symptom scale short form (VSS-SF) score at 6 weeks after vertigo onset. Secondary outcomes include effects of the intervention on activities of daily life, mood/anxiety, vestibular function recovery (vestibulo-ocular reflex gain and saccades), and mobility measures. The full list of outcome measures is provided in Table 3 below.

**Table 3:** Outcome measures

Level of outcome	Outcome measures
Primary outcome	The vertigo symptom scale short form (VSS-SF) score at 6 weeks after vertigo onset.
Secondary outcomes	The between-groups mean dizziness handicap inventory (DHI) score at 6 weeks and 3 months after vertigo onset.
	The between-groups changes in timed 25-foot walk test (T25-FW) and timed balance tests from baseline to 6 weeks and 3 months; the between groups body sway during standing and walking (measured by a mobile phone placed on the hip); the time duration for each test and the mobility (number of steps) at 6 weeks and 3 months after vertigo onset.
	The between-groups changes in video head impulse test (vHIT, site-dependent) measured lateral canal VOR gain and saccades from baseline at 6 weeks and 3 months after vertigo onset.
	The between-groups mean vertigo symptom scale short form (VSS-SF) score at 3 months and 12 months after vertigo onset, and the between-group pedometer-derived number of steps walked since last visit at 6 weeks and 3 months after vertigo onset.
	The between-groups mean number of weekly training sessions at 6 weeks.
	Health economic effects on all levels of care (primary, specialized) and society (sick leave).
	The added value from using a multi-sensor movement analysis system to receive multi-joint kinematic output during 25-foot-walk and balance tests, in comparison to using a mobile phone placed on the hip to receive center-of-mass kinematic output.
	The reliability and validity of the Swedish VSS-SF translation.
	The frequency (percentage) of participants with BPPV at 3 months after AVS onset; symptoms indicating BPPV 6 weeks after an AVS using a BPPV-specific questionnaire; positional nystagmus (non-BPPV) 3 months after an AVS; positional vertigo (non-BPPV) 3 months after an AVS; BPPV in the treatment vs control group; and the DHI/VSS-SF/steps/safety results differences between the BPPV, suspected BPPV and non-BPPV groups.

The VSS-SF measures the frequency of 15 vestibular symptoms on a scale from 0 (no symptoms) to 4 (symptoms most days) during the past month. The score range is 0–60 and a difference from baseline of ≥3 points indicates a clinically significant change [10, 11]. The original English version of the VSS-SF has previously been translated and culturally adapted into several languages including Norwegian, Turkish, and Japanese [19–21]. In this study, we have translated the VSS-SF into Swedish using a similar forward/backward translation method and will subsequently test the reliability and validity of the Swedish VSS-SF questionnaire. For the evaluations at week 6 and after 3 months, the time frame (1 month) for the symptoms will be kept the same. However, for the baseline measurement at day 0, participants will be asked to report their symptoms within the last 24 h, and we will use a modified version of the VSS-SF where the response alternatives are adapted to capture the short-term status of participants after vertigo onset. The modified VSS-SF data will be used for the baseline symptom intensity assessments only.

The DHI investigates the effects of dizziness on everyday living. The between-group mean DHI score will be compared at each time point as detailed in the statistics section. The scale has subcategories (functional, emotional, and physical) and these will be analyzed separately as exploratory analyses, although the total scores will be used for the secondary outcomes. The maximum score is 100 points, divided into 32, 40, and 28 points for the functional, emotional, and physical subcategories, respectively. The higher your score, the more dizziness-related problems you have. A total of 16-34 points denotes a mild handicap, 36–52 points denotes a moderate handicap, and 54 points or more denotes a severe handicap. The original version [22] has been translated and adapted to several languages, including Swedish [23], which will be used in the present study.

EQ-5D is a generic instrument comprising five sub-domains (mobility, self-care, usual activities, pain/discomfort, and anxiety/depression) with three severity levels (no, moderate, and severe problems) [24]. In the current study, the Swedish version of EQ-5D-3L will be applied, and the Swedish value set based on experienced health states will be applied for obtaining a health utility score in order to calculate quality-adjusted life years (QALYs) [25]. The UK MvH value set will be applied for sensitivity analyses [26].

The T25-FW data will be collected by using the mean (seconds) of two, timed attempts at walking a clearly marked 25-foot course. Basic body sway data will be collected by using a locally developed application that utilizes cell phone built-in motion tracking. An enhanced gait analysis with a wearable motion tracking system will be performed at the University Hospital of Umeå for in-depth measurements [27, 28]. The motion tracking and gait data consist of sagittal and frontal plane angles and angular velocities from the head, upper body, and legs. The planes are plotted against each other, and large deviations (quartiles or standard deviations) indicate poor balance. An artificial intelligence paradigm to analyze gait recovery will also be explored [29].

The total number of steps walked will be collected from a pedometer provided to each participant and collected at 6 weeks and 3 months after inclusion.

The VOR gain in each participant is expressed as the mean of ≥5 approved impulses for each lateral canal (right, left); on an aggregated level expressed as the median of VOR means and the changes from baseline at 6 weeks and 3 months will be compared between the study arms (affected side). BPPV will be investigated by asking questions regarding paroxysmal motion-dependent vertigo at 6 weeks and 3 months and performing Dix-Hallpike and head roll testing at 3 months.

### Participant timeline {13}

**Fig. 1:**
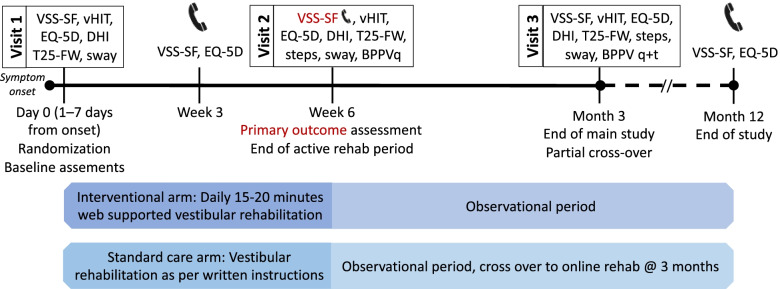
Schematic diagram of the present study—Internet-based vestibular rehabilitation versus standard care after acute onset vertigo: a study protocol for a randomized controlled trial—including the time schedule for enrollment, interventions, and observational periods. Abbreviations: VSS-SF, vertigo symptom scale short form; vHIT, video head impulse test; EQ-5D, EuroQol five-dimension scale; DHI, dizziness handicap inventory; T25-FW, timed 25-foot walk test; BPPV, benign paroxysmal positional vertigo; sway, body sway measurements; q, questions; t, testing

Figure 1 demonstrates the study outline. Partial cross-over occurs at 3 months after enrollment, where participants in the standard care study arm receive access to the online vestibular rehabilitation tool. The schedule of events is further demonstrated in Table 4 below.

**Table 4:** Schedule of events

Visit/contact	Baseline	3 weeks (by phone)	6 weeks	3 months	12 months (by phone)	Comments
Target day	0	21 +/−3	42 +/−3	90 +/−7	360 +/−14	
Eligibility screening, history	x					
Randomization	x					
VSS-SF	x	x	x	x	x	Primary endpoint, at 6 weeks
EQ-5D	x	x	x	x	x	
DHI	x		x	x		
T25-FW	x		x	x		
Body sway	x		x	x		
Enhanced gait and body sway analyses	x		x	x		At selected sites only
vHIT	x		x	x		At selected sites only
Steps, falls, and fractures			x	x		Total steps, since enrollment or last visit
BPPV questions			x	x		
BPPV testing				x		
Compliance		x	x			Questionnaire data, number of weekly sessions
Trial termination form (local)				x		Last site visit, at 3 months
Trial termination form (central)					x	
Estimated time requirement	150 min	15 min	120 min	120 min	15 min	Total 7 h per participant

*Abbreviations*: *VSS-SF* vertigo symptom scale short form, *EQ-5D* EuroQol five-dimension scale, *DHI* dizziness handicap inventory, *T25-FW* timed 25-foot walk test, *SD* standard deviation, *vHIT* video head impulse test

### Sample size {14}

Three hundred twenty is the required sample size based on previous experimental data from the study on a Dutch version of the online vestibular rehabilitation tool on primary care chronic dizziness patients (mean VSS-SF score at 3 months = 8.1 [SD 7.4] in the intervention group, and 11.5 [SD 9.9]) in the control group) [13]. Assuming a standard deviation of 9 and a correlation between pre and post measurements of 0.55, a sample size of *n*=266 is powered (90% power, 0.05 α) to detect a clinically significant change in VSS-SF score of 3 [10, 11]. Assuming a 20% dropout rate, the sample size needs to be increased to *n*=320 to preserve power. Conservative observational data suggest a yearly incidence of 36 patients with acute onset vertigo and nystagmus per 100,000 inhabitants in the Umeå University Hospital uptake area [5, 30].

### Recruitment {15}

Eligible patients will be identified by the site study coordinator through daily vigilance of the emergency department and hospital wards, including the internal medicine, neurology, and ear-nose-throat (ENT) wards. This includes contacting on-call doctors, the emergency department staff, manually screening patient lists in the relevant wards, and notice-board ads and information brochures at each department and ward. ENT outpatient clinics will identify eligible patients from subacute AVS visits. Each site PI will implement local variations of these procedures so as to identify eligible patients. Our aim is to complete recruitment of a total of 320 participants by December 2023, given an estimated rate of recruitment of approximately 160 participants a year.

## Assignment of interventions: allocation

### Sequence generation {16a}

The randomization sequence is arranged in random block sizes of 4, 6, and 8 with 1:1 allocation using site-specific randomization lists generated in R, and the randomization will be performed by an independent statistician at Registercentrum Norr (GG). As a precaution measure, the lists will contain 4 times the number of posts as the expected number of participants at each site. Randomization will be site stratified. The primary effects analysis will include a random effect for site to control for influence from stratified randomization.

### Concealment mechanism {16b}

The allocation will not be known before interventions are assigned by either the participant or the site study PI/research staff that enrolls the participant since the randomization is revealed in the eCRF and only upon the participant’s enrollment and subsequent registration in the eCRF baseline form.

### Implementation {16c}

The allocation sequence is generated by an independent statistician at Registercentrum Norr (GG). The participants are enrolled by the site study PI or research staff. Once the participant is enrolled and subsequently registered into the eCRF, the allocation is revealed by clicking a button in the eCRF baseline form.

## Assignment of interventions: blinding

### Who will be blinded {17a}

Treatment allocation will be open to participants, site study PIs and involved research staff, while blinded primary endpoint evaluations will be performed by telephone at week 6 by staff whose sole assignment is the blinded primary evaluation at 6 weeks. Data collection will be performed as detailed in the following sections.

To minimize the risk of an increased rehabilitation effect facilitated by the added motivation of participating in a clinical trial (in both study arms), the study staff will be instructed to avoid acting as “coaches” and to put focus on data collection when in contact with participants. Thus, the rehabilitation effect in the intervention arm will be isolated to the vestibular rehabilitation app, whereas the effect in the control arm will be isolated to mimic current clinical practice. Since sites will differ according to patient base (e.g., ENT outpatient clinics versus stroke wards), it entails site-mediated differences in participant characteristics. Any bias stemming from these differences will be mitigated by including a random effect for site in the primary outcome analysis, and by site stratified randomization.

### Procedure for unblinding if needed {17b}

The clinical investigation is only blinded for the evaluators at week 6 and for the statistical analyses. Therefore, no emergency code breaking protocol is needed.

## Data collection and management

### Plans for assessment and collection of outcomes {18a}

The study personnel will be trained in data collecting, entry, and coding by either the sponsor, the PIs, or other research staff as needed.

The Swedish translations of VSS-SF, DHI, and EQ-5D-3L are collected locally at all sites by the site study coordinator. Except for the VSS-SF at 6 weeks (primary endpoint), which will be collected through phone interview by a blinded study coordinator, these questionnaire data will be gathered via in-person interviews at day 0, week 6, and after 3 months. VSS-SF and EQ-5D data will also be obtained by phone interview at week 3 and at 12 months.

The 25-foot walk data (T25-FW) will be collected by having participants walk a clearly marked 25-foot course and recording the mean time out of two attempts. Basic body sway data will be collected using a locally developed application that utilizes cell phone built-in motion tracking. For in-depth measurements of body sway, an enhanced gait and balance analysis with a wearable motion tracking system will be performed at Umeå University Hospital [27, 28].

The balance data will be collected by having participants standing on a balance foam pad with their feet together and folding both arms across their chest for a maximum of 30 s or until fail (i.e., coming off balance by extending either arm out, taking a step on or off the foam pad, or about to fall). The participants will be asked to hold this position under four different conditions: eyes open or closed, with head shakes from side to side or keeping their head still. The timer starts as soon as the participant is in position and the time for each test (30 s or less) will be recorded into the eCRF.

The participants will be provided with a pedometer and instructed to wear the pedometer as often as possible during the first 3 months of the study. The total number of steps in the first 6 weeks, and from week 6 to 3 months will be recorded into eCRF and will be compared between the study arms at week 6 and at 3 months.

At sites with access to vHIT, the VOR gain and catch-up saccades will be measured by performing vHIT at day 0, 6 weeks, and 3 months. The catch-up saccades on the affected side (yes/no, proportion of impulses with saccades) will be compared between the study arms, at 6 weeks and 3 months after vertigo onset.

To capture potential long-term effects of early intervention, the Umeå University Hospital study coordinator will contact all participants via telephone at 12 months after inclusion and collect one final round of VSS-SF and EQ-5D data.

The clinical investigation ends when the last participant has completed the last follow-up. The sponsor will notify the Swedish Medical Products Agency within 15 days of the end of the clinical investigation.

### Plans to promote participant retention and complete follow-up {18b}

Dropout from the study including the cause for the discontinuation where given is documented in the eCRF system's trial termination form, and any future data collection will be halted. Withdrawal or declining to further participate in the study will not affect their medical care.

### Data management {19}

Each participant in the study is assigned a unique study identification number. All participants are registered in a participant identification list (participant enrollment and identification list) that ties the participant's name and personal number to a study identification number. All data will be registered, managed, and stored in a way that ensures correct reporting, interpretation, and verification.

An in-house designed eCRF (REDCap, https://projectredcap.org) is used to collect data for the study and the information is kept on a secure dedicated server. Except for site-specific screening lists and participant identification lists, which will be preserved, managed, and archived in a way that allows for accurate reporting, interpretation, and verification, all study site data will be collected using REDCap. This also applies for the EQ-5D-3L questionnaires (interview, paper format), which are used to collect primary data before data entry into REDCap. The eCRF system logs and timestamps all user activity.

A data monitoring unit (see below) will conduct an eCRF review every 6 months to ensure that the study data is complete, and missing data queries will be directed to the site PI. Any outstanding queries will be resolved as above after the last participant has completed the 12-month follow-up phone call, followed by database lock according to the REDCap policy at Umeå University.

If any part of the data is handled by any other organization, whether inside or outside the EU, appropriate agreements and/or other documentation will be established, to ensure that the data processing is carried out in accordance with the provisions of the General Data Protection Regulation (EU ordinance 2016/679, GDPR) and other relevant legislation, before any data transfer takes place.

The content of the informed consent form complies with all applicable integrity and data protection laws. The participant will be provided detailed information about how their study data will be collected, used, and published in the participant information and informed consent form. The study data will be stored in accordance with national data legislation, as explained in the participant information and informed consent form. Data is stored on a secure dedicated server at Umeå University and is maintained by Umeå University’s central IT unit and backed up on a regular basis.

### Confidentiality {27}

All information processed by the sponsor will be pseudonymized and assigned to a study ID.

All data collection at study sites will be done using the eCRF REDCap, a system with an audit trail to prevent unauthorized data changes. Access to REDCap is approved by the site PI and granted by the study organization at Umeå University, and each log-in requires two-factor authentication to ensure the integrity of participant information. Site-specific screening lists and participant identification lists will be managed in a safe manner and stored in a locked cabinet with restricted access.

Authorized representatives of the sponsor, as well as relevant authority, may require access to parts of medical and/or study records that are relevant to the study, including the participant’s medical history, for verification of the data, as explained in the informed consent form. Sponsor representatives must be approved by the person responsible for the medical records at the health care institution and sign a confidentiality agreement to gain access to the participant’s medical records. The records may not leave the premises of the health-care facility. In the event of a data security breach, the established routines for each affected organization will be followed to minimize the repercussions.

The PIs and sponsor will maintain the essential clinical investigation documents in the investigation site files archive and sponsor files archive, respectively. The sponsor must maintain all documentation and data for at least 10 years after the rehabilitation tool’s removal from the market. All local study documentation will be archived by the PIs for at least 10 years, or the length of time stipulated by the local institution.

### Plans for collection, laboratory evaluation, and storage of biological specimens for genetic or molecular analysis in this trial/future use {33}

This trial does not involve collecting biological specimens for storage.

## Statistical methods

### Statistical methods for primary and secondary outcomes {20a}

The primary analysis will be performed using the intention to treat cohort on both primary and secondary outcomes. As part of the sensitivity analysis, per-protocol analyses (PP) will also be undertaken. Descriptive statistics will be used to describe baseline characteristics in the two study arms. Both study arms will be described and compared at baseline using a table including relevant baseline variables. In accordance with the CONSORT protocol, no statistical tests of the baseline values will be performed.

Two-sided statistical tests will always be performed. The primary outcome will be analyzed using analysis of covariance (ANCOVA) adjusting for baseline measurements; known predictors of the outcome including diagnostic group, sex, age, and baseline symptom intensity in order to gain precision in treatment effect estimates; and inclusion of a random effect for site to control for influence from stratified randomization. The primary endpoint results at 6 weeks will be presented as the ANCOVA-generated adjusted VSS-SF difference with 95% confidence interval and p-value between the study arms. The VSS-SF has previously been analyzed in a randomized trial assuming normality [13]. If a clear deviation from normality is detected, an ordinal logistic regression ANCOVA will be used for the primary outcome.

For secondary outcomes with repeated measurements, we will utilize linear mixed models analysis for continuous outcome variables, and binary logistic mixed effects models for binary outcome variables with random effects for individual and site. These methods can accommodate for repeated measurements within an individual as well as imbalances caused by missing data. The intervention effect will be modeled using a time by intervention interaction term.

We will also perform statistical validation of the Swedish translation of the VSS-SF questionnaire, including analyses of reliability (test-retest reliability and internal consistency reliability) and validity; construct validity (convergent and discriminant validity) by investigating item-to-item associations between VSS-SF/VSS-SF domains and DHI; and criterion validity by correlating the domains to DHI and balance tests. For the test-retest study, the first 100 included participants will complete the evaluator-blinded 6-week assessments of the VSS-SF twice, 48 h apart, via telephone [19].

The intervention effects on T25-FW and VOR gain and saccades will be evaluated using ranked analysis of covariance (ANCOVA), which gives robust estimates despite the presence of extreme outliers in skewed data.

We will present crude and adjusted analyses, controlling for baseline values of the investigated outcome as well as the prespecified predictors of sex, age, education level, number of chronic diseases, and baseline AVS etiology. For an overview of data collection by time points, please see the schedule of events below (Table 5). The statistical analysis plan will be managed by a statistician at Registercentrum Norr (GG).

**Table 5:** Aim-specific statistical analysis plan

Analysis	Individuals analyzed	Sample size	Estimated difference based on previous results mean (SD)	Power	Method
Between group mean VSS-SF difference at w3, w6, m3, and m12	All participants	160+160	8.1 (7.4) vs 11.5 (9.9) [13]	94%	ANCOVA
Between group mean EQ-5D difference until m3	All participants	160+160	Unknown	Unknown	Mixed models
Between group mean EQ-5D difference at 12m	All participants	160+160	Unknown	Unknown	ANCOVA
Between group mean DHI difference until m3	All participants	160+160	24.4 (20.8) vs 29.2 (21.1) [13]	54%	Mixed models
Mean of means T25-FW (seconds) and timed balance tests (seconds) until m3	All participants	160+160	Unknown	Unknown	Mixed models
Between group mean difference of means of vHIT gain (affected side) at w6 and m3	Participants at sites with access to vHIT	80+80	0.52 (0.24) vs 0.69 (0.25)^a^ [31]	99%	Ranked ANCOVA
Proportion with catch-up saccades on vHIT (affected side) at w6 and m3	Participants at sites with access to vHIT	80+80	Unknown	Unknown	Binary logistic regression
Body sway until m3	All participants	160+160	Unknown	Unknown	Mixed models
Enhanced gait and body sway analysis until m3	Umeå participants only	25+25	Unknown	Unknown	Mixed models
Total steps since last visit at w6 and m3.	All participants	160+160	Unknown	Unknown	ANCOVA
Self-reported vestibular rehabilitation compliance until 6w (mean number of training sessions)	All participants	160+160	Unknown	Unknown	Mixed models
Proportion with falls or fractures	All participants	160+160	Unknown	Unknown	Binary logistic regression
Proportion with BPPV	All participants	320	Estimated frequency 10%	N/A	Binary logistic regression

Abbreviations: *VSS-SF* vertigo symptom scale short form, *EQ-5D* EuroQol five-dimension scale, *DHI* dizziness handicap inventory, *T25-FW* timed 25-foot walk test, *SD* standard deviation, *vHIT* video head impulse test, *w6* 6 weeks, *m3* 3 months, *m12* 12 months

^a^Power calculations based on mean gain differences before and after vestibular rehabilitation

### Interim analyses {21b}

A drop-out rate analysis will be undertaken once 50% of the included participants have completed the 6-week primary endpoint examination. If the drop-out rate exceeds 20%, the 320-person inclusion objective may be increased in order to maintain power.

### Methods for additional analyses (e.g., subgroup analyses) {20b}

Subgroup analyses will be conducted by searching for interaction between:

- The baseline diagnostic group (categorical), i.e., vestibular impairment (most commonly vestibular neuritis), cerebrovascular vertigo (stroke), and vertigo NOS (dizziness not otherwise specified).
- Sex (categorical)
- Age (continuous)
- Study site (categorical)
- Symptom severity at baseline – VSS-SF (continuous)
- BPPV diagnosis at 3 months or not (categorical)

### Methods in analysis to handle protocol non-adherence and any statistical methods to handle missing data {20c}

To preserve the ITT principle for the primary endpoint, we will use (1) the 6-week VSS-SF assessment, (2) the 3-week assessment, and (3) the 3-month evaluation, in that order. A sensitivity analysis will be performed by comparing the primary analysis results with and without the imputed values from the 3 weeks and 3 months values.

To test the robustness of the data, we will exclude the 10% of participants in each study arm with the lowest numbers of self-reported weekly training sessions.

Any significant variations from the initial statistical analysis plan as outlined will be disclosed to the medical products agency.

### Plans to give access to the full protocol, participant level-data and statistical code {31c}

To maximize outreach, the study group will aim to make the tool available upon completion of the study provided the primary endpoint results are favorable. The details regarding this will be established during the study and presented in conjunction with the final study report to be submitted to the Swedish Medical Products Agency within 1 year of the study’s conclusion.

## Oversight and monitoring

### Composition of the coordinating center and trial steering committee {5d}

The trial's administrative center is the University Hospital of Umeå, which hosts administrative and research staff that coordinate the experiment, keep track of its progress, and provide administrative support to all the study locations involved. The trial is overseen by a trial steering committee (TSC), comprising the trial sponsor (JS), the Umeå study PI (SS), the Sundsvall study PI (EF), representatives from the engineering staff (HG and FÖ), the local study nurse, and two research expert nurses employed by the Clinical Trials Unit (CTU). The TSC provides informational mailings to all site study PIs and research staff at the participating sites at least four times a year, video-conference meetings at least biannually, and invites all study personnel to annual on-site meetings in Umeå.

### Composition of the data monitoring committee, its role, and reporting structure {21a}

An independent monitor will monitor the study before the study begins, during the study conduct, and after the study has been completed, to ensure that the study is carried out according to the CIP and that data is collected, documented, and reported according to ISO 14155:2020 and applicable ethical and regulatory requirements. The purpose of monitoring is to verify that the participant's rights, safety, and well-being are met, and to ensure the data in the CRF is complete, correct, and consistent with the source data, as specified in the study's monitoring strategy. A separate risk-based monitoring strategy lays out the specifics of the monitoring operations.

### Adverse event reporting and harms {22}

Adverse events (AE) or adverse device effects (ADE), including serious adverse events (SAE) or serious adverse device effects (SADE) as well as device deficiency (DD) will be continuously monitored throughout the study. The rehabilitation tool is considered equivalent to “Device” in the context of the current investigation.

The PI or an authorized designee will record all AEs in the eCRF except for the following: vertigo, nausea or mild discomfort during the vestibular rehabilitation, regardless of randomization allocation as these symptoms are common after acute vertigo and during vestibular rehabilitation and all participants are thus expected to develop such symptoms; falls and fractures, which are recorded and reported as stand-alone endpoints and therefore not included in the AE reporting. Moreover, all SAEs, all DDs, and any new findings in relation to the above-mentioned events will also be recorded in the eCRF.

The PIs will notify the sponsor immediately, but no later than 3 days after the investigation site study personnel become aware of an AE/SAE. The PIs will report events to the sponsor using the eCRF AE/SAE form. All of the following will be reported to the Swedish Medical Products Agency (Läkemedelsverket) by the sponsor: any SAE that has a causal relationship with the investigational device or investigation procedure, or where such a causal relationship is reasonably possible; any DD that could have resulted in an SAE if appropriate action had not been taken, intervention had not occurred, or circumstances had been less fortunate; and any new findings in relation to any event above.

The sponsor will submit reports by completing the “Summary Reporting Form” (MDCG 2020-10/1). For each reportable event or for new findings to previously reported events, the form will be filled in or updated respectively. Events that signify an imminent risk of death, serious injury, or serious illness and thus necessitate prompt remedial action will be reported immediately, and no later than two calendar days after the sponsor becomes aware of a new reportable event or new information in relation to an already reported event. Any other reportable events or new findings or updates to it will be reported immediately, and no later than 7 calendar days after the sponsor becomes aware of the new reportable event or new information in relation to an already reported event.

The care provided in this study is conducted by the regions and the participants are thereby protected by the Swedish Patient Insurance provided by LÖF.

### Frequency and plans for auditing trial conduct {23}

During the study, an independent monitoring team from CTU at the University Hospital of Umeå audits each participating site approximately once a year. The first audit occurs early in the trial, after five participants have been recruited and randomized, and no later than 4 months after the first participant the site has been randomized, whichever comes first. At this point is it also verified that the trial is being conducted in line with the protocol, ISO 14155:2020, and other applicable regulations. If the monitor or the sponsor notices inconsistencies, more frequent monitoring may be necessary following discussion with the site. Additional central monitoring is also performed at least annually in order to regularly monitor eCRF data quality and completeness. After the last participant has had their last follow-up in the trial, a “close-out” audit will also be performed at each site. After each audit, an audit report is produced and sent to the sponsor and to the site-PI. Should there be need for corrections, a re-check is scheduled at a later time to ensure that all requests for updates and corrections are met and that note to files are submitted where necessary.

### Plans for communicating important protocol amendments to relevant parties (e.g., trial participants, ethical committees) {25}

Any study protocol amendments that are deemed as substantial modifications will be submitted to the Swedish Medical Product Agency (Läkemedelsverket) and to the Swedish Ethical Review Authority (Etikprövningsmyndigheten) and await approval before implementation. After approval the modifications are immediately communicated to all concerned parties (including site PIs), and if there are amendments that affect the participation of the participants, the participants will be asked for new consent under the new conditions.

### Dissemination plans {31a}

The study is registered at ClinicalTrials.gov, a publicly available database, and the content is updated as the clinical investigation progresses, and the results will be entered at the trial completion. Regardless of the outcomes, a clinical investigation report will be written within 1 year of the completion of the clinical trial or within 3 months in the event of its early termination or temporary suspension. A plain language summary of the research findings will also be prepared. The clinical investigation report and summary will be submitted to the Swedish Medical Products Agency.

Within 2 years of the clinical study's completion, the sponsor, in collaboration with the site PIs, will develop and submit a scientific report of the clinical investigation's findings for publication in a scientific journal. The site PIs are invited to participate as co-authors in the publication of the primary endpoint (expected highest impact publication) and may also participate in additional publications based on interest and contribution, as agreed. The ICMJE authorship criteria will be applied, and all participating PIs have agreed to take part in the interpretation of the results and the drafting of the primary outcome report.

## Discussion

This is the first study that assesses the effects of an online vestibular rehabilitation tool on vestibular rehabilitation in participants with acute vestibular syndrome and which uses a Swedish translation of VSS-SF as its primary outcome. The study will investigate, using a hospital-based cohort study, the effectiveness of the online tool in preventing chronic vertigo, dizziness, and balance disturbances in patients with acute onset vertigo compared to standard care in the form of written instructions. Furthermore, the clinical investigation is designed such that the standard care study arm will be granted access to the online rehabilitation tool at the end of the core study at 3 months, meaning that if the online tool works better than standard treatment, all study participants will benefit from it. Following the completion of the trial, we aim to make the tool publicly available throughout the country through healthcare channels. Many dizzy patients in Sweden could benefit from a freely available, easy, and efficient vestibular rehabilitation tool, with improved quality of life and ability to work as a result.

## Trial status

Currently recruiting. The first participant was recruited in October 2021. Our aim is to complete recruitment by December 2023, with the final 12-month follow up in December 2024.

Clinical investigation plan (CIP): version 1.4, approved 2022-01-19

Study Start Date: October 2021

Estimated Recruitment Completion: December 2023

Estimated Study Completion Date: December 2024

## Data Availability

The sponsor has full access to, and retains all rights to, the data generated in the study. The site PIs participate as co-authors in the publication of the primary endpoint (expected highest impact publication) and may also participate in additional publications based on interest and contribution, as agreed. The sites will also receive reimbursement for study participant inclusion and follow-up as detailed in the Site Agreement.

## Abbreviations

ADE: Adverse device effect
AE: Adverse event
AVS: Acute vestibular syndrome
CIP: Clinical Investigational Plan
DD: Device deficiency
CTU: Clinical Trials Unit
DHI: Dizziness handicap inventory
eCRF: Electronic Case Report Form
EQ-5D: EuroQol five-dimension scale
ICMJE: International Committee of Medical Journal Editors
ISO: International Organization for Standardization
ITT: Intention-to-treat (including all data from all individuals who have participated in the study)
LVFS: Läkemedelsverkets författningssamling (English: Swedish Medical Products Agency’s statutes)
LÖF: Landstingens Ömsesidiga Försäkringsbolag (English: the Swedish patient insurance provider)
PI: Principal Investigator
PP: Per Protocol analysis (including only data from participants who have completed the study completely in accordance with the CIP, with no deviations from the CIP)
SADE: Serious adverse device effect
SAE: Serious adverse event
SD: Standard deviation
T25-FW: Timed 25-foot Walk Test
TSC: Trial Steering Committee
VSS-SF: Vestibular Symptom Scale Short Form
vHIT: Video head impulse test
VOR: Vestibulo-ocular reflex
